# Mode-locking via dissipative Faraday instability

**DOI:** 10.1038/ncomms12441

**Published:** 2016-08-09

**Authors:** Nikita Tarasov, Auro M. Perego, Dmitry V. Churkin, Kestutis Staliunas, Sergei K. Turitsyn

**Affiliations:** 1 Aston Institute of Photonic Technologies, Aston University, Birmingham B4 7ET, UK; 2 Institute of Computational Technologies SB RAS, 630090 Novosibirsk, Russia; 3 Departament de Física i Enginyeria Nuclear, Universitat Politècnica de Catalunya, Colom 11, 08222 Terrassa, Spain; 4 Novosibirsk State University, 1 Pirogova St., 630090 Novosibirsk, Russia; 5 Institució Catalana de Recerca i Estudis Avançats (ICREA), Pg. Lluis Companys, 23, 08010 Barcelona, Spain

## Abstract

Emergence of coherent structures and patterns at the nonlinear stage of modulation instability of a uniform state is an inherent feature of many biological, physical and engineering systems. There are several well-studied classical modulation instabilities, such as Benjamin–Feir, Turing and Faraday instability, which play a critical role in the self-organization of energy and matter in non-equilibrium physical, chemical and biological systems. Here we experimentally demonstrate the dissipative Faraday instability induced by spatially periodic zig-zag modulation of a dissipative parameter of the system—spectrally dependent losses—achieving generation of temporal patterns and high-harmonic mode-locking in a fibre laser. We demonstrate features of this instability that distinguish it from both the Benjamin–Feir and the purely dispersive Faraday instability. Our results open the possibilities for new designs of mode-locked lasers and can be extended to other fields of physics and engineering.

## Abstract (web summary)

Dissipative Faraday instability by periodic zig-zag modulation of spectral losses can lead to pattern formation in variety of systems. Here, the authors employ this nonlinear instability as a technique to achieve high-order harmonic mode-locking in a fibre laser.

## Graphical abstract

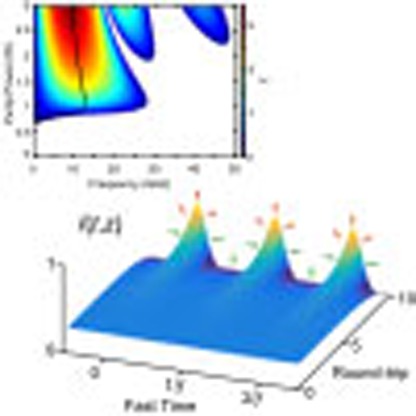

Understanding the mechanisms underlying generation of coherent structures from noise or uniform field distribution is a fundamental problem of nonlinear science and its numerous practical applications ranging from biology to astrophysics. Formation of coherent patterns originates in nonlinear instabilities that can often be described within generic mathematical models^1^, and, as a result, are very similar across different fields of science. Modulation instability is responsible for the symmetry breaking of homogeneous spatio-temporal states or wave envelopes and the formation of stable patterns in a variety of physical media. There are three major and well-known classes of instabilities. First, Benjamin–Feir (BF) instability, originally introduced in fluid dynamics^2^,^3^, and later demonstrated in a variety of physical systems^2^,^4^,^5^. Second, Turing instability^6^, where the combined action of local self-enhancement and lateral inhibition due to the interplay between nonlinearity and diffusion in coupled equations leads to the pattern formation in chemical, biological systems^7^,^8^, as well as in nonlinear optics where diffraction or dispersion substitute diffusion^9^,^10^,^11^. Third and finally, Faraday instability, which results from the periodic in time modulation of a dispersive parameter of the system^12^ and was studied in different systems, ranging from vertically shaken granular media^13^ to periodically driven spatially extended chemical systems^14^, repulsive (defocusing-type) Bose–Einstein condensates^15^,^16^ and nonlinear optics^17^,^18^. Recently, a new dissipative type of Faraday instability was demonstrated theoretically^19^, in the framework of the complex Ginzburg–Landau equation, where a suitable parametric modulation of spectral losses can lead to pattern formation. The dissipative Faraday instability, induced by periodic modulation of spectral losses, differs substantially from the usual Faraday instability where a dispersive parameter, diffraction, dispersion or nonlinearity, is periodically modulated. Although the traditional Faraday instability can also occur in a dissipative system, such as an externally driven optical resonator, as it has been predicted theoretically^18^,^20^, and recently demonstrated experimentally^21^, both the excitation method and the sidebands growth process differ from the case where a dissipative element is modulated.

In nonlinear science, instability of a uniform state in non-equilibrium systems triggers a transition to new states with a rich variety of spatio-temporal structures. In engineering, instabilities are often associated with somewhat undesirable effects and problems that ‘should be avoided'. However, they can also play a constructive role in technology, defining device design and control methods in non-equilibrium systems. For instance, instabilities might be important for seeding, enhancing certain frequencies, dumping others and leading to the formation of stable patterns, with the characteristics of emerging structures determined at the nonlinear stage^18^,^20^,^22^,^23^.

There is a great practical demand for devices and physical mechanisms, which break the symmetry of the uniform or continuous wave state of the laser radiation leading to the formation of temporal structures—optical pulses. Usually, in laser systems, the symmetry breaking is achieved through modulation instability^22^,^23^, the introduction of a modulator or a saturable absorber. The latter can be either material, such as for instance carbon nanotubes^24^ and SESAM^25^, or an effective one based on physical propagation effects, such as, for example, nonlinear polarization evolution^26^, Kerr lens^27^, nonlinear optical loop mirror^28^ and others. The demand for a new controllable and stable all fibre mode-locking mechanism is driven by the field of high power mode-locked fibre lasers, where it has a great practical value.

Here we experimentally demonstrate the recently theoretically predicted dissipative Faraday instability^19^ in a simple configuration Raman fibre laser. The induced instability leads to high-order harmonic mode-locking with tunable repetition rate and high environmental stability. The experimental results are in a good agreement with theoretical predictions and numerical simulations.

## Results

### Dissipative Faraday instability

**Figure 1:**
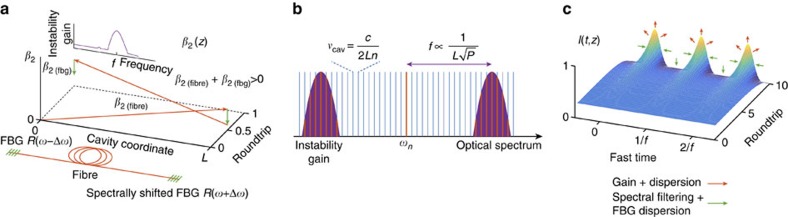
Dissipative Faraday instability in a fibre laser. (**a**) The light propagating in a linear laser cavity experiences periodic modulation of group velocity dispersion and spectrally dependent losses. In the particular example of dispersion modulation, the normal dispersion accumulated along the propagation over the fibre length is partially compensated at the cavity mirrors. The zig-zag spatial modulation of the dissipation with spatial frequency *k*=2*π*/Λ, where Λ=2*L*, excites the dissipative Faraday instability. The instability frequency is related to half of the spatial forcing frequency*, k/*2 (parametric resonance condition), via the dispersion relation *ω*(*k*). (**b**) The Faraday instability gain developed in the system couples the phases of each optical cavity mode *ω*_*n*_ and cavity modes separated by the instability frequency *f*=*ω*(*k*)/2*π*. (**c**) Coupling of modes separated by frequency *f* leads to the harmonic mode-locking and pattern or pulse train formation in the temporal domain. At the later stages, the shape of the pulses is defined by the combination of self-similar propagation and spectral filtering. FBG, fibre Bragg gratings.

The instability is initiated by the introduction of a periodic spatial modulation of a dissipative parameter of the system (Fig. 1a). When parameters of a system are modulated with the longitudinal period Λ, corresponding to the spatial frequency *k*=2*π*/Λ, then the Faraday instability is initiated, with the first unstable mode oscillating with the wavenumber *k*/2, that is, the double period. The corresponding pattern forms in the temporal domain, with the characteristic frequency *ω* related to the wavenumber *k*/2, via the nonlinear dispersion relation *ω*(*k*). In the proposed configuration, the light travelling in the cavity experiences periodic spectral losses after reflection from the spectrally shifted mirrors at each end of the cavity (zig-zag-like spectral filtering). The reflection profiles of mirrors were shifted in spectral domain by +Δ*ω* and −Δ*ω*.

It has been shown theoretically^19^ that such a configuration, with frequency detuned mirror reflectivity profiles, leads to the dissipative Faraday instability. Indeed, when losses are applied to the spectral region +Δ*ω*, the damped spectral components experience sudden growth, gaining energy due to a four wave-mixing process where the homogeneous mode and the symmetrically located modes in spectral region −Δ*ω* are involved. Then, after nonlinear evolution of the electric field inside the fibre laser, when the losses are applied in the spectral region −Δ*ω*, the process repeats symmetrically; and the periodic iteration of such a scheme drives an average growth of spectral sidebands—modulation modes—which eventually destabilizes the homogeneous state of the system.

In a fibre laser resonator, the instability gain resulting from the modulation of spectral losses, with the maximum at the frequency *f*=*ω*(*k*)/2*π*, couples the phases of the longitudinal cavity modes (Fig. 1b), leading to the formation of a pattern in the temporal domain, with the period 1/*f*. At the nonlinear stage of evolution, the pattern in this dissipative system is supported by the balance of Kerr nonlinearity, gain saturation, dispersion, spectrally dependant loss and gain (Fig. 1c).

### Experimental results

To demonstrate experimentally the possibility of exciting a stable temporal pattern of optical pulses by dissipative Faraday instability we built a linear cavity fibre laser with spectrally shifted fibre Bragg gratings at both ends providing the modulation of dissipation in a zig-zag manner (Fig. 1a). The system is described by the generalized nonlinear Schrödinger equation^29^,^30^ (see also Methods) with a loss coefficient having spatial and spectral dependence: *α* (*z*, *ω*). Unlike the case of BF instability, the growth of the spectral sidebands is not due to the average effect and is not continuous, but synchronized with the period of modulation^19^. In the temporal domain, the pattern forms with the period corresponding to the strongest component in the gain spectrum.

In the experiment we used a 2.2 km-long fibre placed between two highly reflective mirrors—fibre Bragg gratings (Supplementary Fig. 1). The fibre was chosen with high normal group velocity dispersion, *β*_2_=25.5 ps^2^ km^−1^, so BF instability is not initiated. Laser mirrors had negative linear chromatic chirp of −53 ps^2^, partially compensating the fibre dispersion, so that the total cavity dispersion was normal. The gratings had super-Gaussian reflectivity profile with a full-width at half-maximum of 1 nm, and were shifted by ∼0.75 nm or 90 GHz, and stabilized by Peltier elements. The laser operated via the Raman gain, and generated at 1,550 nm, pumped by a multi-Watt power quasi-continuous wave pump laser at 1,450 nm. Polarization was not controlled in any way.

**Figure 2:**
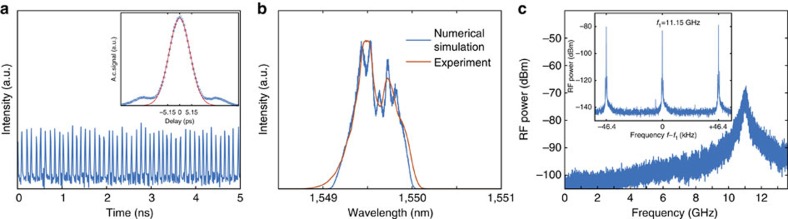
Experimental results. (**a**) Pulse train and intensity autocorrelation of a single pulse (inset) with a Gaussian fit. (**b**) Optical spectrum of the pulses in experiment (orange) and in numerical simulations (blue). (**c**) Typical radio-frequency (RF) spectrum (resolution bandwidth 10 kHz) with inset showing the 11 GHz peak in detail (resolution bandwidth 1 Hz). Intermodal distance corresponds to the cavity length.

With the properly chosen parameters of the system, the laser readily mode-locked as soon as the lasing threshold was reached, and produced a train of high-repetition-rate pulses (Fig. 2a), with no stable continuous wave regime of operation observed, due to the presence of the instability. The generated pulses, propagating in the normal dispersion cavity, evolve asymptotically into parabolic pulses^31^ with the optical spectra broadening by a factor of two (Supplementary Fig. 2 and Supplementary Note 1), before the shifted gratings reshape them and suppress the background radiation through a mechanism known as Mamyshev regenerator^32^,^33^. The pulses had the uncompressed full-width at half-maximum of 7.3 ps measured after reflection from a grating (Fig. 2a, inset), and were Gaussian shaped, which is consistent with the expected asymptotic shape of the output of a Mamyshev regenerator. Optical spectrum had a width of 0.65 nm or 80 GHz, and is shown in Fig. 2b. Power spectrum (Fig. 2c) reveals large supermode noise-associated timing jitter in the system, with repetition rates reaching 11 GHz, which corresponds to the ∼2.4 × 10^5^ harmonic of the fundamental frequency.

It should be noted that no additional technical efforts were made to improve the quality of mode-locking, however, even in such simple configuration the laser operation was environmentally very stable and reproducible. Therefore, there is a great potential for further improvement in the quality of mode-locking based on the proposed instability.

### Theoretical analysis

**Figure 3:**
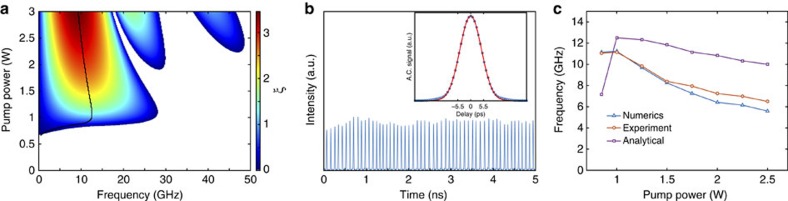
Faraday instability spectrum and pulses repetition rate. (**a**) The parametric resonance tongues of the Faraday instability are revealed by the Floquet linear stability analysis, which takes into account the growth process of perturbations in modulated systems over one modulation period and allows the calculation of the frequency-dependent growth exponents (instability spectrum). At variance with what happens in the BF instability, the frequency of the most unstable mode is a decreasing function of the pump power and this is a genuine feature of parametric instabilities in the net normal dispersion regime. The gain maximum is emphasized by the black line. (**b**) The results of numerical simulations. Pulse shape and repetition rates are in a good agreement with the experiment. (**c**) The scaling of repetition rate with power for the numerical simulations, experiment and the prediction of the stability analysis.

The onset of Faraday patterns can be studied using the linear Floquet stability analysis and direct numerical simulations of the set-up. Figure 3a shows the gain level dependence on power obtained from Floquet linear stability analysis, with spectral detuning of the chirped cavity mirrors taken into account. As expected, for Faraday (both dispersive and dissipative^19^) instability, in the case of net normal dispersion, the frequency corresponding to the maximum gain decreases with the pump power increase and follows the simple asymptotical dependence: *ω*_inst_∝*P*^−1/2^ in contrast to the BF instability. The pulse repetition rate dependence on power from the numerical simulation (Supplementary Note 2) agrees well with the experiment (Fig. 3c), and is consistent with the predictions of the linear Floquet stability analysis.

Although it has been shown that periodic group velocity dispersion modulation can lead to instability and pattern formation^18^,^21^, we emphasize that the instability leading to generation of pulses in our system is triggered by the periodic modulation of dissipation. While the dispersion compensation, operated by the chirped gratings has a small impact on the pulses' repetition rate and is not responsible for exciting the instability. This point is discussed in detail in Supplementary Note 3 where it is shown that the gain related to the modulation of dispersion, when modulation of dissipative terms is turned off, is located at a much higher frequency compared with the case where dissipation is modulated (Supplementary Fig. 3) so that it can be ruled out as the main mechanism of the instability. Furthermore, we have verified that the repetition rate of the pulses is only marginally affected by varying drastically the amount of chirp in the gratings (Supplementary Note 3 and Supplementary Fig. 4). Further studies should be undertaken to exploit the dispersion compensation for the control of the pulses' chirp and shape.

## Discussion

In conclusion, we predict that the dissipative Faraday instability-initiated patterns, originally proposed in the very general framework of the complex Ginzburg–Landau equation, can be observed in a Raman fibre laser and we demonstrate it experimentally. The result is a train of pulses with extremely high repetition rate, corresponding to the instability frequency. The proposed and designed dissipative Faraday instability in a fibre laser resonator constitutes a novel nonlinear science-based mechanism of mode-locking with potential for various practical applications. In particular, the formation of a pattern of optical pulses in a dissipative system is promising for the generation of high-repetition-rate pulses with energies unachievable in a conservative system.

In our experiments the main focus was on presenting the novel mechanism of mode-locking by keeping the set-up design as simple as possible to highlight the underlying physical processes. Despite being extremely simple, the laser readily mode-locks, once in the range of corresponding operational parameters. This shows a great potential for control of this mode-locking mechanism and future improvements in the quality of the generated pulse train.

## Methods

### Numerical simulations

To confirm the nature of the mode-locking mechanism, we performed a numerical analysis using a well-established model describing a Raman fibre laser^30^. We used the Ikeda-map-like procedure: in the first stage an integration of the fields evolution equations along the fibre followed by the action of the first Bragg grating was performed, a second integration of the fields to describe backward propagation, and interaction with the second grating close the full roundtrip. The equations for the Stokes *A*_s_ and pump *A*_p_ (forward^+^and backward^−^ propagating) waves evolution along the fibre read


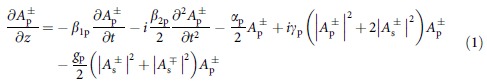



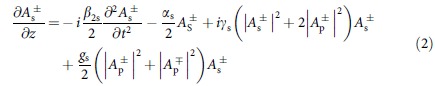


where *z* denotes the propagation spatial coordinate along the fibre, *t* is the time coordinate, *β*_2_ is the fibre group velocity dispersion, *β*_1_ accounts for the group velocity mismatch between pump and Stokes waves, *g* is the Raman gain, *γ* is the Kerr nonlinear phase shift coefficient and *α* describes the distributed losses. For numerical simulations we have used the following set of parameters: *β*_2p_=25.78 ps^2^ km^−1^, *β*_2s_=25.49 ps^2^ km^−1^, *g*_p_*=*2.5 (W·km)^−1^, *g*_s_*=*2.32 (W·km)^−1^, *α*_p_*=*0.6 (km)^−1^, *α*_s_*=*0.6 (km)^−1^ (also takes into account connector, splice, coupling and bending losses), *γ*_p_=8 (W·km)^−1^, *γ*_s_=6.5 (W·km)^−1^ and distance between the two mirrors *L*=2.2 km. Subscripts, p and s, in the coefficients refer to the pump and Stokes fields, respectively. Equations (1) and (2) have been supplemented by the following boundary conditions in correspondence to the cavity extremities: chirped Bragg mirrors having frequency detuned super-Gaussian profiles of sixth order described by the functions *f*_±_=*R* exp[−(*ω*±*ω*_±_)^6^/Ω^6^] with *R*=0.98, *ω*_±_=150 rad ns^−1^ and Ω=190 rad ns^−1^. Pump injection was applied in correspondence to the first cavity mirror while the second cavity mirror was chosen to have no reflectivity for the pump according to the experimental situation. The spectrum of the multimode pump field was simulated as a sum of spectral modes with randomly generated amplitude and phases with a Gaussian envelope.

In the numerical study, first, we calculated numerically the homogeneous field distributions of equations (1) and (2) by suppressing temporal modulations; the stationary state reached corresponds to non-zero field background with the amplitude depending on the value of the pump field injected at *z*=0. Next, we performed both linear stability analysis and a complete integration of the equations for many cavity roundtrips to characterize the full dynamics.

### Stability analysis

The Floquet stability analysis was performed by computing the evolution of real and imaginary small perturbations of each spectral mode over one cavity roundtrip. Diagonalization of the map provided the Floquet multipliers spectrum *F*(*ω*). Denoted by *F*_m_(*ω*)=max (|*F*(*ω*)|) the maximum among the Floquet multipliers absolute values, the average growth exponent for each mode can be calculated as *ξ*=ln (*F*_m_(*ω*)). Note that the power gain in dimensional units of km^−1^ is given by *G*=2*ξ*/Λ. In this way we could draw the instability map in Fig. 3a, which depicts the growth exponent in the *ω*−*P* space, where *P* is the injected pump power.

The linear stability analysis predictions differ slightly from the results of the experiment and from full numerical integration. The small discrepancy is likely due to a nonlinear resonance, which takes place when the amplitude of the growing modulation modes becomes large enough to violate the assumption of the small perturbation (linear regime).

### Data availability

The data that support the findings of this study are available from the corresponding author on request.

## Additional information

**How to cite this article:** Tarasov, N. *et al*. Mode-locking via dissipative Faraday instability. *Nat. Commun.* 7:12441 doi: 10.1038/ncomms12441 (2016).

## Supplementary Material

Supplementary Information Supplementary Figures 1-4, Supplementary Notes 1-3 and Supplementary References
