# Supplementary material for: Mode-locking via dissipative Faraday instability
Source: Nat Commun. 2016 Aug 9;7:12441. doi: 10.1038/ncomms12441 (PMC4980481; doi:10.1038/ncomms12441)
Supplement: Supplementary Information — Supplementary Figures 1-4, Supplementary Notes 1-3 and Supplementary References [file ncomms12441-s1.pdf]

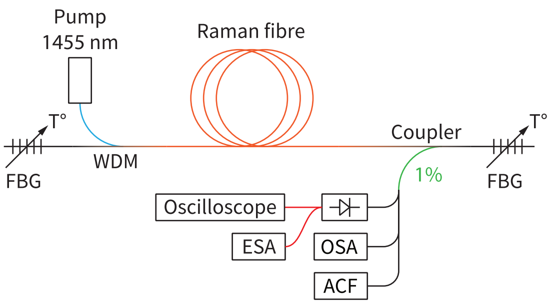

Supplementary Figure 1 | Experimental setup.

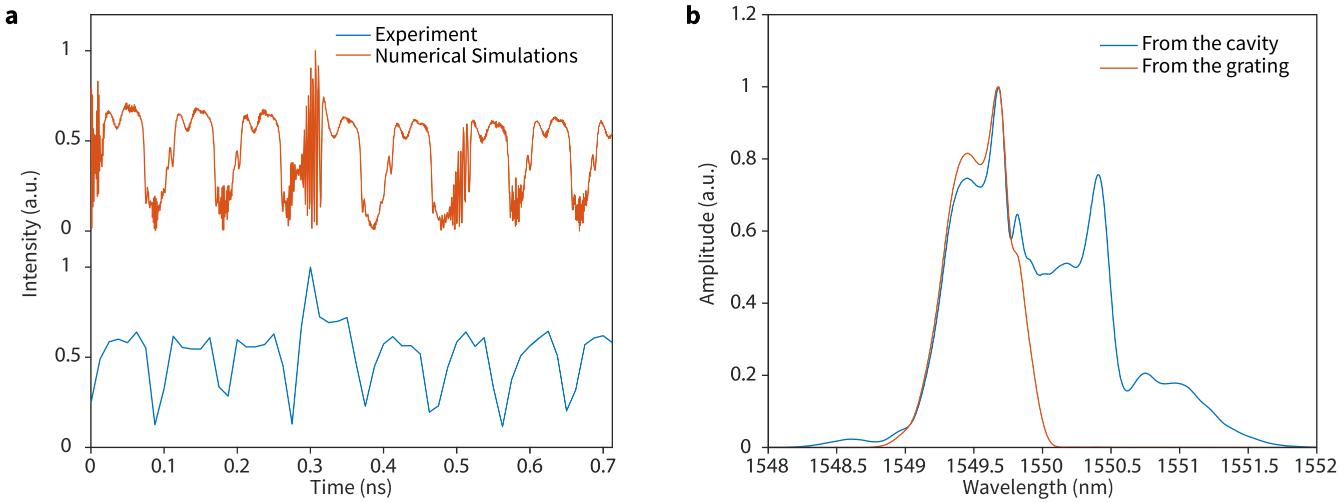

Supplementary Figure 2 | Experimental results. (**a**) The shape of the pulses coming after propagating in the cavity, before being reflected by the output grating. Experimental and numerical simulation results agree well both qualitatively and quantitatively. (**b**) The optical spectrum of the pulses after propagation through the cavity (blue), and after reflection from the grating (orange).

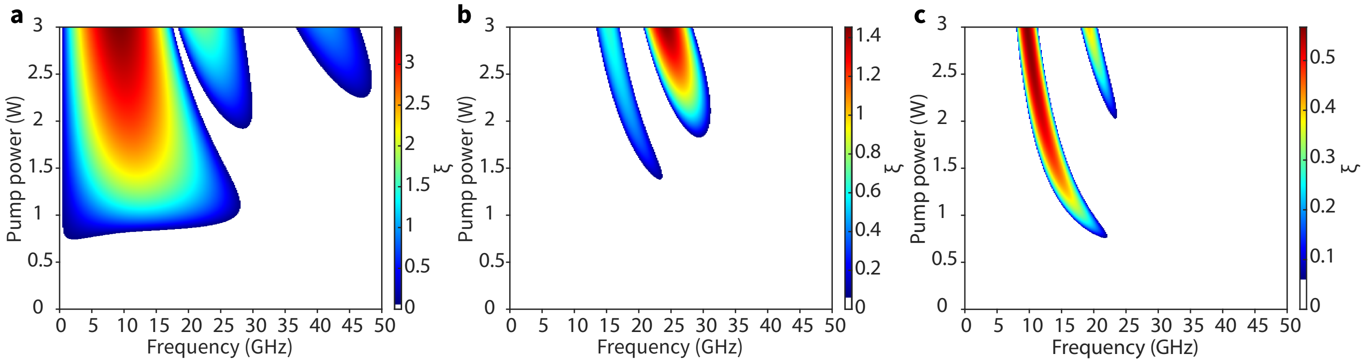

Supplementary Figure 3 | Faraday instability diagrams. (**a**) The instability diagram for the case of detuned gratings, but without dispersion modulation. (**b**) The instability diagram for the case of dispersion modulation with undetuned gratings. (**c**) The instability diagram for the case when neither dispersion or dissipation are modulated.

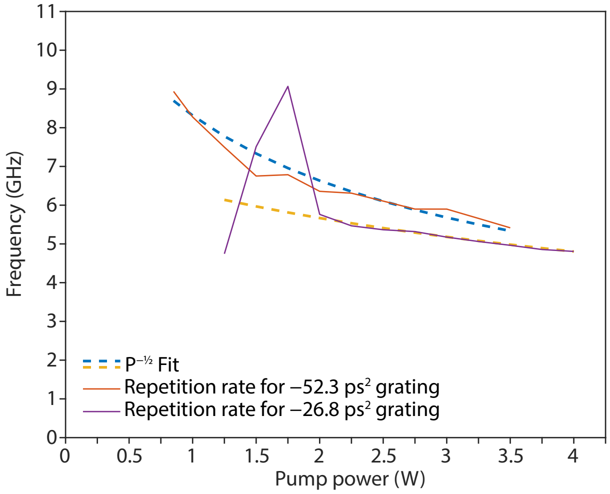

Supplementary Figure 4 | Frequency scaling for different net cavity group velocity dispersions.

Supplementary Note 1 Experimental setup

The schematic diagram of the Raman fibre laser used in the experiment to study the properties of the dissipative Faraday instability is shown on the Supplementary Figure 1. The resonator was formed of 2.2 km of OFS Raman fibre with normal dispersion $D = -20$ ps nm^{-2} km^{-1}, dispersion slope 0.031 ps nm^{-2} km^{-1} at 1550 nm, Raman gain coefficient 2.5 (W·km)$^{-1}$ and high nonlinearity 6.5 (W·km)$^{-1}$. Two identical 1 nm wide fibre Bragg gratings (FBG) with high reflectivity 97%, 3rd order super-Gaussian profile and chromatic dispersion −53 ps^2 were used as cavity mirrors. They were spectrally shifted by approximately 0.75 nm and stabilised by Peltier elements. Pump radiation at 1450 nm was coupled into the cavity with the help of wavelength division multiplexing coupler (WDM). Optical spectrum and intensity dynamics were monitored from the 1% rejection port immediately after reflection from the output grating.

Fast 50 GHz DC-coupled photodetector and 33 GHz real-time oscilloscope were used to register the temporal intensity dynamics. Optical spectra were measured using 0.02 nm resolution bandwidth optical spectrum analyser Yokogawa AQ2670C. Intensity autocorrelator Femtochrome 103-XL was used for auto-correlation function (ACF) measurements. RF spectra were obtained with 13.6 GHz electrical spectrum analyser.

With the gratings spectral shift set by the Peltrier elements, the laser readily mode-locks as soon as the pump level is above the lasing threshold of 0.85 W, and operates stably up to the pump powers 2.5 W. The detuning of the gratings affects both the pulse repetition rate and pulse timing jitter, which agrees well with the prediction given by the linear stability analysis and numerical simulations.

Other combinations of normal dispersion fibres, such as OFS IDF, and FBGs with different width and chromatic dispersion values were also studied both numerically and experimentally, with the results that agree well with stability analysis predictions.

To provide another reference point, the radiation coming from the cavity was registered at the point before the output grating. As expected, the pulses had close to parabolic shape Supplementary Figure 2a, with a characteristic dip in the middle, which, again, was reproduced in the numerical simulations.

The effect of spectral broadening that pulses experience during the propagation in the cavity is demonstrated on the Supplementary Figure 2b, where the spectrum of laser radiation coming from the cavity, and incident on the FBGs (see Supplementary Figure 1), is shown together with the spectrum of the optical pulses registered immediately after reflection by the FBG. Depending on the pump power, the spectrum can broaden by a factor > 2, maintaining nearly a flat top profile, typical for parabolic pulses.

Supplementary Note 2 The scaling of the Faraday Instability

In order to provide an estimation for the functional dependence of the maximally growing mode in the dissipative Faraday instability, we considered the nonlinear Schrödinger equation for the electric field envelope ψ propagating in a fibre with normal group velocity dispersion $\beta_2 > 0$ and Kerr nonlinearity $\gamma > 0$:

$$\frac{\partial \psi}{\partial z} = -i \frac{\beta_2}{2} \frac{\partial^2 \psi}{\partial t^2} + i\gamma |\psi|^2 \psi. \tag{1}$$

The Bogoliubov modes, oscillatory perturbations on top of the homogeneous field background ψ_0, are stable for focussing non-

linearity and normal group velocity dispersion and obey the following dispersion relation:

$$k^2 = \frac{\beta_2 \omega^2}{2} \left(\frac{\beta_2 \omega^2}{2} + 2\gamma \left|\psi_0\right|^2 \right). \tag{2}$$

In presence of the periodic forcing of a system parameter with spatial frequency k, we expect the Bogoliubov modes having wavenumber which is an integer multiple of half of the forcing frequency, to synchronize with the forcing and hence to be excited. From the parametric resonance condition the first excited temporal mode has frequency ω which satisfies the dispersion relation $\omega(k/2)$. For a dissipation modulation having spatial period equal to Λ, we have $k = 2\pi/\Lambda$ and with help of Supplementary Equation 2 in the long wavelength limit we obtain:

$$\omega \approx \frac{\pi}{\Lambda \sqrt{\beta_2 \gamma \left|\psi_0\right|^2}}. \tag{3}$$

Supplementary Equation 3 shows that the instability frequency depends on the inverse of the squared root of the field intensity and provides a phenomenological scaling formula for the systems with parametric modulations. The Raman laser used in our experiment is described by a much more complicated model based on coupled generalised nonlinear Schrödinger equations, where dissipation plays a key role and the influence of cross-phase modulation, spatial nonuniformity of the gain and group velocity mismatch between pump and Stokes fields are not negligible. Nevertheless, Supplementary Equation 3 provides the basic functional dependence on the laser pump power. Such a scaling can be obtained for dissipative systems described by the Complex Ginzburg-Landau Equation as well [1].

Supplementary Note 3 Instability diagrams and dispersion management

We stress the fact that the dispersive Faraday instability does not introduce substantial modifications to the main tongue of the instability spectrum. In the case of pure dissipative modulation (unchirped gratings) the instability spectrum Supplementary Figure 3a, is identical to that of Figure 3a of the main paper where both dispersive and dissipative modulation are present. If the gratings were chirped but not detuned in frequency (purely dispersive modulation) we obtained the instability spectrum depicted in Supplementary Figure 3b. The tongue responsible for dispersive Faraday instability, located around 25–30 GHz, occurs at about twice the frequency of the first dissipative Faraday instability tongue simply due to the fact that the modulation period of dispersion is half of the modulation period of dissipation and this leads to a twice higher instability frequency as shown by Supplementary Equation 3.

The small gain and lower frequency tongue in Supplementary Figure 3b, is caused by the periodic power variation experienced by the Stokes field due to the pumping which takes place only on one side of the resonator and hence with half of the group velocity dispersion modulation frequency.

Indeed if neither dissipation, nor dispersion are modulated — considering unchirped and not detuned mirrors — only the tongue due to periodic power variation and its second harmonic survive, as depicted in Supplementary Figure 3c. When chirped mirrors are used, the second instability tongue due to periodic power variation overlaps with the first tongue induced by group velocity dispersion (GVD) modulation (Supplementary Figure 3b): in that case the two effects enforce themselves.

We stress that both situations corresponding to Supplementary Figure 3b and Supplementary Figure 3c do not generate any pulsations in our system. It is important to comment that, even though the periodicity of the dissipation and of the pump depletion induced periodic power variation are the same, the tongue induced by the zig-zag modulation of the dissipation has a different and characteristic shape "attached" to the "zero" frequency as it is clearly depicted in Figure 3a of the main paper and in Supplementary Figure 3a; and it is the only one that can explain the periodic pulsation observed in the system's dynamics.

We emphasize what already mentioned in the main paper: dispersion management is necessary in the experiment in order to stabilise the pulse train that otherwise will be less regular and not easily tunable. Such an extra degree of freedom makes the systems much more flexible and versatile and provides a key tool for mode-locked lasers design. The compensation of the accumulated dispersion by the gratings also provides a convenient mechanism of pulse compression before the pulses are out-coupled from the cavity. The impact of the dispersion modulation on the instability frequency and pulse repetition rate, however, is not very large. On the Supplementary Figure 4 the frequency scaling for two different sets of gratings is shown. The fibre dispersion at the Stokes wavelength was the same in both cases $\beta_{2s} = 56.1$ ps^2, while the dispersion of the gratings differed by a factor of 2.

We have checked both numerically and experimentally that the purely dispersive Faraday instability was not able to excite any regular pulsation in our system most likely due to the highly noisy nature of the Raman laser, and the absence of a suitable saturable absorber or other pulse reshaping mechanism.

Supplementary References

[1] Perego, A. M., Tarasov, N., Churkin, D. V., Turitsyn, S. K. and Staliunas, K. 'Pattern generation by dissipative parametric instability'. *Phys. Rev. Lett.* **116,** 28701 (2016).
